# Outdoor Localization Using BLE RSSI and Accessible Pedestrian Signals for the Visually Impaired at Intersections

**DOI:** 10.3390/s22010371

**Published:** 2022-01-04

**Authors:** Kiyoung Shin, Ryan McConville, Oussama Metatla, Minhye Chang, Chiyoung Han, Junhaeng Lee, Anne Roudaut

**Affiliations:** 1 RSS Center, Korea Electrotechnology Research Institute, Ansan 15588, Korea; kyshin@keri.re.kr (K.S.); mhchang@keri.re.kr (M.C.); 2 Department of Computer Science, University of Bristol, Bristol BS8 1TR, UK; om16384@bristol.ac.uk; 3 Department of Engineering Mathematics, University of Bristol, Bristol BS8 1TR, UK; ryan.mcconville@bristol.ac.uk; 4 Corporate Affiliated Research Institute, Human Care Co., Ltd., Ansan 15258, Korea; gorns1200@empas.com (C.H.); human2@humancare.co.kr (J.L.)

**Keywords:** visually impaired, localization at an intersection, pedestrian navigation, BLE RSSI

## Abstract

One of the major challenges for blind and visually impaired (BVI) people is traveling safely to cross intersections on foot. Many countries are now generating audible signals at crossings for visually impaired people to help with this problem. However, these accessible pedestrian signals can result in confusion for visually impaired people as they do not know which signal must be interpreted for traveling multiple crosses in complex road architecture. To solve this problem, we propose an assistive system called CAS (Crossing Assistance System) which extends the principle of the BLE (Bluetooth Low Energy) RSSI (Received Signal Strength Indicator) signal for outdoor and indoor location tracking and overcomes the intrinsic limitation of outdoor noise to enable us to locate the user effectively. We installed the system on a real-world intersection and collected a set of data for demonstrating the feasibility of outdoor RSSI tracking in a series of two studies. In the first study, our goal was to show the feasibility of using outdoor RSSI on the localization of four zones. We used a k-nearest neighbors (kNN) method and showed it led to 99.8% accuracy. In the second study, we extended our work to a more complex setup with nine zones, evaluated both the kNN and an additional method, a Support Vector Machine (SVM) with various RSSI features for classification. We found that the SVM performed best using the RSSI average, standard deviation, median, interquartile range (IQR) of the RSSI over a 5 s window. The best method can localize people with 97.7% accuracy. We conclude this paper by discussing how our system can impact navigation for BVI users in outdoor and indoor setups and what are the implications of these findings on the design of both wearable and traffic assistive technology for blind pedestrian navigation.

## 1. Introduction

APS (Accessible Pedestrian Signals) has been developed to help blind and visually impaired (BVI) people cross the road safely. Such systems involve the end-user pressing a button at the crossroads to get information about the traffic. Such information comes in an audible and/or vibrotactile form. Although such systems are widespread, they suffer from some limitations, including the difficulties of locating a traffic signal pole and activating the APS system, and uncertainties around crossing cycles [1].

One way to improve APS is to use walking navigation technologies which mainly use GPS (Global Positioning System) to determine the location of users [2,3,4]. However, this solution has limitations. The standalone GPS determines position with an accuracy of 5 to 10 m [5], but this can go up to 7 to 13 m coverage position error using a Smartphone in an urban environment [6]. In many countries, the width of a single carriageway with two lanes is 5.5 to 7.3 m and the width of a dual carriageway with two lanes is 14.6 m [7,8]. Because the width of these roads is within the margin of error of the GPS, it is not possible to know which sidewalk a pedestrian is on. Additionally, some of the applications required for such systems (e.g., lane-level positioning system, collision warning system and Geographic Information System (GIS)) require far better accuracy than that given by the standalone GPS [9].

Another solution is to use Bluetooth, such as Bluetooth Low Energy (BLE), for positioning. Such systems have started appearing in a few countries such as Korea [10,11], France [12], and the US [13]. The pedestrian using a remote control, that pairs with a Bluetooth beacon using Bluetooth Low Energy (Bluetooth 4.0), placed at the beginning and end of a crossing, can know when to cross the road. The estimated distance from each nearby beacon is calculated by analyzing the Received Signal Strength Indicator (RSSI) from the network of beacons that are deployed at the given location. One way to measure the distance using RSSI is to calculate the distance in meters within the radius of the Bluetooth beacon [14]. However, although such a system can be efficient in the case of a single road crossing, it becomes more challenging for the end-users in the case of complex road architectures that are typical in larger cities. In such cases, it can be challenging for pedestrians to identify which Bluetooth beacon the signal comes from, potentially leading to unsafe crossings. In order that BVI people can cross an intersection, more information is required by the system, such as recognizing the location of the intersection, recognizing the direction of pedestrians, and recognition of pedestrian signals [15].

In this paper, we studied a method to overcome the problems of the Bluetooth system to enable accurate positioning outdoors. We experimentally verified various features with k-Nearest Neighbors (kNN) and Support Vector Machine (SVM) classifiers, which are among the most widely used machine learning classifiers, and various RSSI window sizes for moving average filter to reduce the noise of RSSI, discovering which method results in the best performance. An initial survey with end-users shows indeed that a technology that has a short response time would be beneficial, thus motivating our use of RSSI Bluetooth. After presenting our system we investigate how to tune our algorithm in a series of two studies. In the first study, we divided an intersection into four zones to test the feasibility. We achieved 99.8% accuracy using kNN with the features being the average values of each RSSI over a sliding 3 s window for moving average. In a second study, we investigated an additional machine learning classifier, the SVM, the features of which performed best when classifying the location of the person at the intersection within nine zones. We installed the system at a real-world intersection and collected data. We achieved 97.7% accuracy using an SVM with the features being the average values of each RSSI, the standard deviation, median, and IQR using a 10-point moving average. Thus, we found that the area where a BVI person is located can be detected with high accuracy in this way, using our Crossing Assistance System (CAS) measured through a smartphone. Through this, it is possible to deploy the APS to provide core information to BVI people for walking navigation by calculating their location information.

In summary, our contributions are: (1) an improvement of APS that localizes a pedestrian using RSSI Bluetooth outdoors; (2) An initial survey demonstrating that BVI pedestrians need a rapid response for such scenarios; (3) a series of two studies to tune the algorithm showing we can detect the location of people with 97.7% accuracy at a real-world intersection; (4) a discussion on considerations to to move this work further and design implications for wearable and traffic assistive technology for blind navigation/intersection crossing.

## 2. Related Works

### 2.1. Accessible Pedestrian Signals

**Figure 1:**
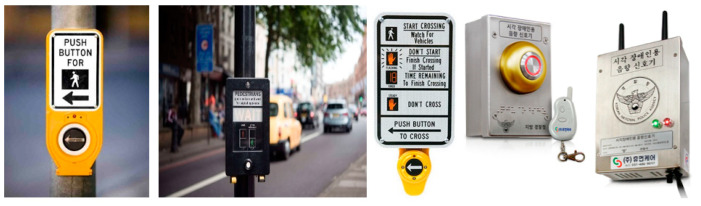
Examples of pushbutton-integrated accessible pedestrian signals from various manufacturers.

Accessible pedestrian signals (APS), as shown in Figure 1, let pedestrians who are blind or visually impaired know precisely when the walk interval begins by providing audible and/or vibrotactile information coinciding with visual pedestrian signals [16].

Barlow et al. reports the results of research on crossings by blind pedestrians at complex signalized intersections, before and after the installation of APS with innovative audible beaconing features, designed to improve wayfinding. They report APS decreased the delay in starting to cross, increased the number of crossings that participants began independently and within the walk interval, increased the number of crossings that were completed before the signal changed, and reduced the number of requests for assistance [15].

A mobile-based personal APS, named Mobile Accessible Pedestrian Signals (MAPS), was proposed by Liao [17]. The main function of the MAPS system was to provide BVI people with the available intersection geometry condition as well as signal timing information through a smartphone application. Using built-in sensors of a smartphone (e.g., GPS and digital compass) along with signal phasing and timing plans, the MAPS can inform pedestrians not only when to cross, but also how to align with the crosswalk. We build on finding from the MAPS system by validating this approach in the real world.

Kim et al. proposed a BLE-enabled APS that can enable two-way communication via Bluetooth with a smartphone [10]. By using the connectionless communication method utilizing the advertisement mode of BLE 4.0, it is possible to receive signals from the APS with multiple Bluetooth connections to enable multiple access from the smartphone. In a situation where multiple smartphones and BLE devices are mixed, using the BLE function, the user selects only the desired audible signal. However, these previous studies are limited to monitoring the status of traffic lights or replacing push buttons with smartphone applications through improved APS and pedestrians’ smartphones.

Existing APS with built-in Bluetooth uses RSSI to calculate only how many meters a pedestrian is within a radius of the APS and activates the nearest APS with a smartphone. However, in practice, an error occurs because the RSSI value is affected by the metal pole of the traffic light [18]. Additionally, this approach does not detect the location of BVI pedestrians’ navigation, but rather wirelessly replaces the push of a button by hand.

### 2.2. Localization System with Smartphone

Many navigation systems for BVI people have used smartphones because smartphones have numerous sensors, such as accelerometers, gyroscopes, magnetometers, proximity sensors, GPS, microphones, barometers, cameras, and Time of Flight (ToF) sensors and connectivity technologies, such as Wi-Fi, Bluetooth, Near Field Communication (NFC) and Cellular Connectivity [19].

GPS is frequently used for localization. Jafri and Ali [20] proposed a system that allows users to record a customized path to a particular destination based on personal considerations whether the unevenness of the terrain or the absence of hazards, such as traffic intersections. Velázquez et al. [4] presented a wearable navigation system for BVI pedestrians that combines a GPS and tactile-foot stimulation for information presentation.

However, due to limited coverage by GPS, complementary systems are needed to keep track of users along their route [21]. This has been done with the addition of vision [22] or Inertial Measurement Unit (IMU) sensors [23], for example. But image processing technology still requires a lot of computing power, and there is a problem of installing or holding a camera that can look ahead. The IMU sensor using dead reckoning requires an additional process to remove the accumulated error.

While GPS is mostly used outdoors, Bluetooth is used indoors where GPS is not available [24,25,26,27]. After installing several Bluetooth beacons, the method calculated the user’s position using the fingerprint method [27], triangulation [26] method, etc. Since the width of the intersection is within the error range of the GPS, it is not easy to know exactly which sidewalk BVI pedestrians are on. This calls for the need to investigate the use of BLE RSSI at the intersection to increase the accuracy of localization for the visually impaired.

### 2.3. RSSI-Based Bluetooth Analysis

Positioning or localization technology using Bluetooth Low Energy (BLE) RSSI has been actively studied until recently in an indoor environment that GPS does not cover [28,29,30,31,32]. Indoor positioning methods usually require a dataset of collected RSSI data and the associated labeled position. This dataset is called the reference set, or the fingerprint database. Then, the methods estimate the current user position using the knowledge from the reference set. Some popular indoor positioning techniques use machine learning algorithms such as Hidden Markov Models (HMMs), kNN, SVM, and Deep Neural Networks (DNN) [30] to perform this estimation automatically and accurately.

The set of RSSI values measured from each beacon is used to train the machine learning algorithm, while the output of the model is predicted location. Since the RSSI value is sensitive to the surrounding environment, e.g., due to the movement of people or vehicles, noise-canceling methods such as the Kalman filter [33], particle filter [34], Mean and Median filter [29], and the moving average filter [35] are typically used to improve performance.

## 3. CAS System Implementation

**Figure 2:**
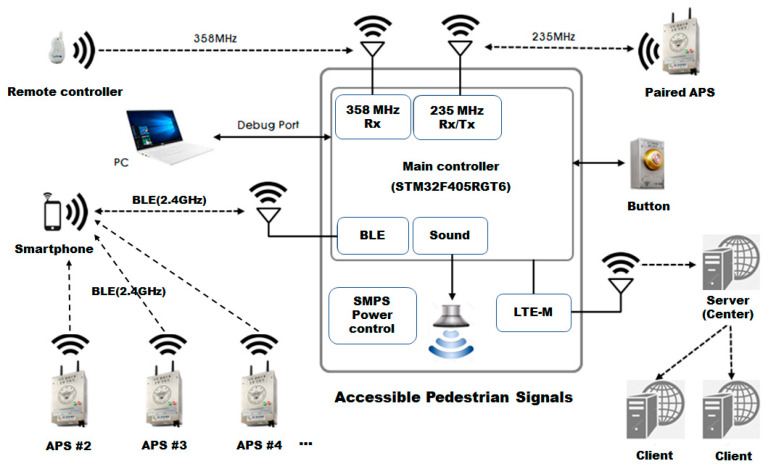
Communication block diagram of Crossing Assistance System (CAS).

In this section, we present our Crossing Assistance System implementation. Each APS installed at an intersection has 235 MHz and 358 MHz RF communication, BLE communication module, and LTE-M communication module as shown in Figure 2. For BVI people in Korea, 235 MHz and 358 MHz are the frequencies allocated for guidance signals. For the remote control, 358 MHz is used for remote operation of the sound signal device, and 235 MHz is the frequency allocated for paired APS. In addition, APS includes a Bluetooth communication module and LTE-M communication module. The Bluetooth communication module is used for communication where a BVI user operates an acoustic signal with a smartphone, and for determining their location by measuring the BLE RSSI value of each APS. The LTE-M module is used to monitor the status of the APS and transmit the status to the remote server.

**Figure 3:**
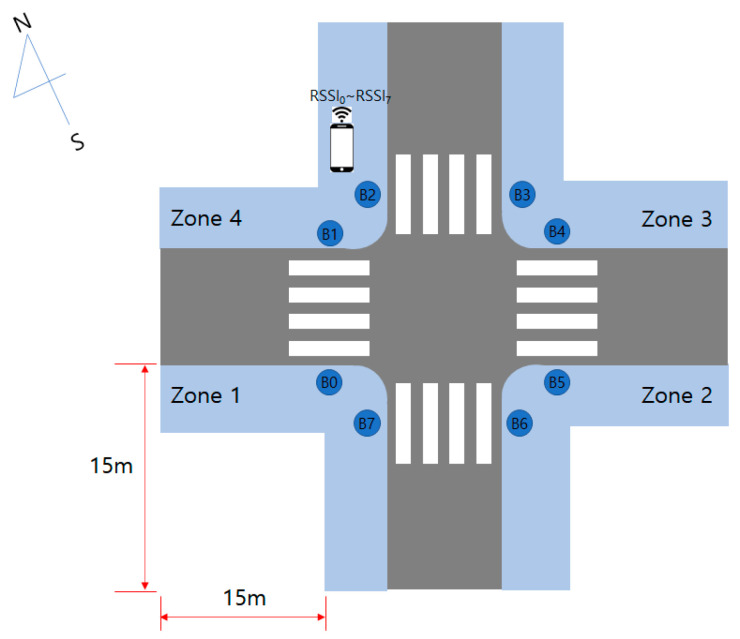
Zone division for the feasibility study.

Eight APSs were installed at the crossroads as shown in Figure 3. Each APS transmits a beaconing signal every 0.5 s, and the smartphone is implemented to receive 2 RSSI signals per second from 8 APSs. An APS with a built-in Bluetooth module uses an omni-directional antenna, but there is a problem that the RSSI value becomes inaccurate even at a short distance due to radio wave interference of the traffic signal pole.

## 4. Study 1: Feasibility on Four Zones

We conducted a feasibility study to verify whether it is possible to locate BVI people on crosswalks at real-world intersections. In this feasibility study, four pedestrian zones were studied initially since there are normally four pedestrian zones and four to five crossing zones at intersections. We tested whether it is possible to detect the location of a pedestrian through RSSI values received from eight Bluetooth beacons, using machine learning.

### 4.1. Method

In the experiment, APS with Bluetooth module was installed in 8 places at the crossroads as shown in Figure 3. The area of each zone for RSSI value collection is 15 × 15 m, and the shape of the zone looks like an ‘L’ because data were collected from the pedestrian path except for the building. The width of the walkway varies depending on the environment but ranged between 2 to 4 m. The Bluetooth beacon was set to transmit an RSSI value every 0.5 s, and RSSI data were acquired for 5 min in each zone using a smartphone (Galaxy A31, Samsung). To acquire data in the test size, one researcher held a smartphone in his hand and moved to cover as many areas as possible in each zone. The average walking speed of a BVI person at a crosswalk is 0.94 m/s [36]. RSSI data was measured after practicing a walking speed to allow a BVI person to move 15 m in about 16 s.

Since the acquired RSSI data contains noise due to vehicles passing through the crossroads, the M-point moving average of the data was calculated as shown in Equation (1):

(1) $$y\left[n\right]=\frac{1}{M}\overset{M-1}{\underset{k=0}{\sum }}x\left[n-k\right]$$

where *M* is the window size. The eight RSSI values calculated in this way were used as features for the kNN classifier. The value of *K* was chosen as the square root of *N*, the total number of points in the training data set. The kNN classifier was chosen as it is one of the most straightforward classifiers in machine learning yet can perform well on many tasks. The measured data was divided into five random sets, and three sets were used to train the model, one was used for verification, and the remaining was used for testing. Multi-class accuracy is defined as the average number of correct predictions as shown in Equation (2):

(2) $$accuracy=\frac{1}{N}\overset{\left|G\right|}{\underset{k=1}{\sum }}\overset{}{\underset{x:g\left(x\right)=k}{\sum }}I\left(g\left(x\right)=\hat{g}\left(x\right)\right)$$

where *I* is the indicator function, which returns 1 if the classes match and 0 otherwise. Signal processing and classification algorithms were implemented using Matlab R2021a.

### 4.2. Results

**Figure 4:**
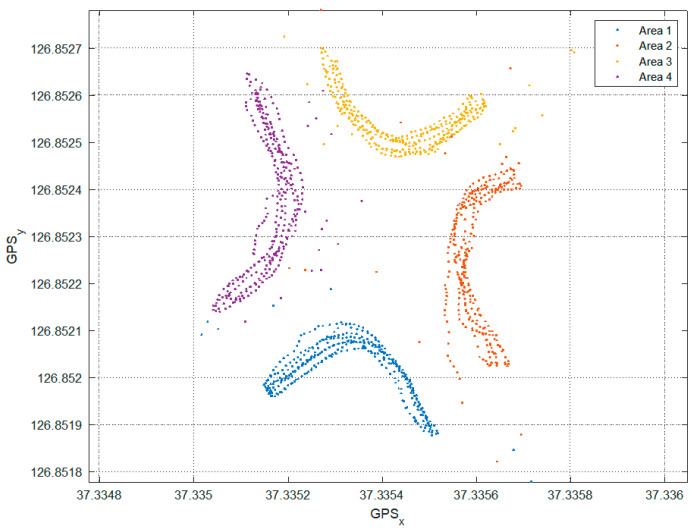
Data acquisition area is drawn by the GPS of the smartphone.

The values measured through the built-in GPS of the smartphone are shown in Figure 4, which also shows points stamped outside the four zones due to the GPS error. X-axis and y-axis represent latitude and longitude respectively.

First, we calculated the accuracy according to the number of points of the moving average. The accuracy of the system varies depending on the number of points in the moving average window. The accuracy was 91.9% for three points, 97.35% for four points, 98.98% for five points, and 99.8% for six points.

**Figure 5:**
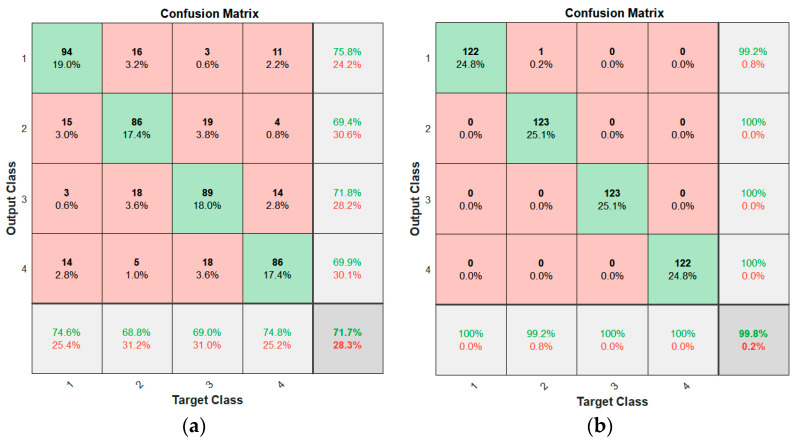
Confusion matrices for (**a**) raw data and (**b**) six points moving average of the Received Signal Strength Indicator (RSSI).

Secondly, we compared the accuracy when the 6-point moving average was calculated and when it was not. When a classification model was trained using raw data without the moving average of the measured RSSI values, the accuracy was 71.7%, and when a classification model was trained with moving average data, the accuracy was 99.8% as shown in Figure 5. Classes 1 to 4 of the x-axis and y-axis represent Zones 1 to 4 in Figure 5. According to this result, we see that the accuracy increased by around 20% when the moving average was used. This demonstrates the importance of noise filtering from the raw RSSI data for outdoor localization.

### 4.3. Questions Raised from the Feasibility Study

When the intersection was divided into four zones centered on the pedestrian path through the feasibility experiment, the zone where the pedestrian was located could be distinguished with high accuracy through the RSSI values of eight beacons. In the development of pedestrian navigation for the BVI people, there is a limit to identifying the location of pedestrians by dividing the intersection into four zones. Therefore, a question is raised about the viability of such a system for more complex scenarios, and about the evaluation of the system in a more challenging setup.

Additionally, a waiting time of 3 s was required to obtain classification results. But, for pedestrian navigation at the intersection, it is necessary to classify the intersection into more than four zones, which may take longer for the algorithm to compute. Thus, the second question arising concerns the window size of the moving average required. The window size is directly related to the waiting time required for the user to obtain the result.

Given these two questions, we supplemented our contribution with an online user survey (Section 5) to receive feedback on how long users are likely to wait for the classification results in this setting. With those results, we studied (Section 6) a more complex setup where we used a total of nine zones including the crosswalk zone. We then tested whether the location of pedestrians could be identified.

## 5. Online Survey

We surveyed how long users are likely to wait to ensure accuracy of the algorithm. The response speed of the smartphone to the user’s location is a very important factor. However, it takes some time for the RSSI to accumulate to increase the accuracy of the location recognition algorithm. Based on the results of this survey, we determined the maximum allowable time required to recognize the user’s location. The scope of the survey is BVI people living in large cities in Korea. The data collected through the survey included gender, age group, visual impairment, type of visual impairment, smartphone use, average daily walking time, APS use, and the maximum allowable waiting time when location recognition accuracy is 70–80%, 80–90%, and 90–100%. The survey was conducted online with the cooperation of the KBU (Korea Blind Union) for a total of 232 BVI people. Of these, 132 respondents were analyzed, excluding those who did not fill out a waiting time survey because they did not use APS.

The results of the survey are shown in Table 1. When the accuracy is 70–80%, 16.7% of people answered that it could not be used, and the largest number of respondents said that they can wait up to 5 s. When the accuracy is 80–90%, 8.3% of respondents said that it cannot be used, and 30.3% of respondents said that it can wait up to 5 s. Even with 90–100% accuracy, 29.5% of respondents reported that they could wait up to 5 s, followed by 3 s. It is interesting that with an accuracy of 90–100%, 10.6% of respondents said they could wait up to 10 s and 18.2% of respondents wanted results in less than a second. Since the low accuracy is not of practical help to BVI people and there was no “can’t use” feedback response, we set the target accuracy as 90–100% and set the target within 5 s, which was the highest frequency in overall accuracy.

## 6. Study 2: Nine Zones Classification

In this study, we tested for a more complex scenario to evaluate the system in a more challenging setup. Navigation for BVI pedestrians, as well as sighted pedestrians, could be improved by providing guidance based on the exact location of the user, especially at intersections. BVI people can only cross the street if they have information about pedestrian signals on the crosswalk in the direction they want to go. Further, when crossing the road, the guidance voice or vibration feedback should be different depending on whether the user is on the crossing or completed the crossing. Therefore, in this study, we used a total of nine zones: four pedestrian zones and four crosswalk zones at intersections, and one zone for diagonal intersections. As with prior studies, eight APSs with built-in Bluetooth were installed at intersections to study whether a pedestrian’s current locations could be identified through smartphones. In the feasibility study, the kNN classifier and moving average values of eight RSSIs were used as features. To classify nine zones, however, in this study, we want to further study the choice of the classifier by also including an SVM, and feature extraction methods beyond taking the average of the RSSIs.

### 6.1. Method

**Figure 6:**
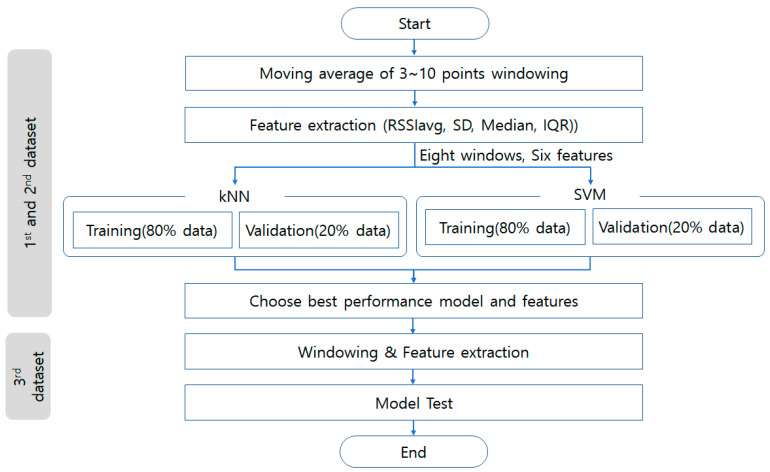
Flow chart of the method used to collect the data.

We selected kNN and SVM classifiers as they are among the most widely used methods for classification. For feature extraction, we calculated the average RSSIs, standard deviation, median, and IQR of each RSSI in an overlapped window as features. A total of six features sets were used: moving average, moving average + standard deviation, median, median + IQR, moving average + median, moving average + standard deviation + median + IQR. The number of points for the moving average was set from three to ten to derive the initial result within five seconds because the smartphone can receive 2 RSSI measurements per second from each APS. The value k of kNN is typically chosen as the square root of N, the total number of points in the training data set [37], and we used the Euclidean distance metric. We selected SVM binary learners and one-vs-one strategies for multi-class classification in SVM using an error-correcting output codes model. kNN and SVM were implemented using the fitcknn and fitcecoc functions provided in MATLAB (MATLAB R2021a, MathWorks). All algorithmic procedures were implemented offline on a standard computer. We trained and validated a total of 96 model combinations (two models, six different sets of features, and eight windows for moving average), and selected the model with the highest accuracy on a validation set. To conduct a fair evaluation of this model on unseen data, we used a new data set, collected on a different day to the training and validation data, to evaluate the performance of the final system. The flow chart of this study method is shown in Figure 6.

**Figure 7:**
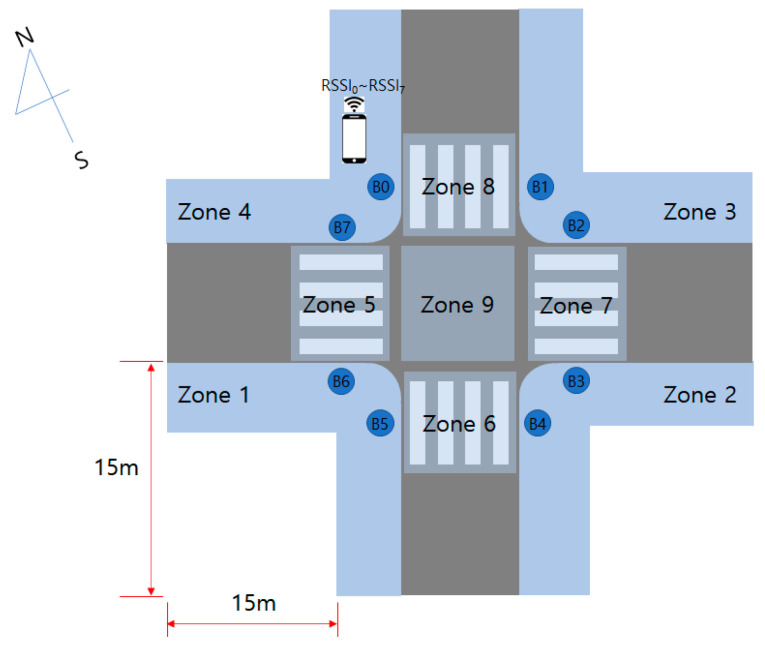
Nine zones for position classification.

In the experiment, APS with a Bluetooth module was installed in eight places at the crossroads as shown in Figure 7. The data was collected by one researcher moving around nine zones with a smartphone (Galaxy A31, Samsung, Korea) in an environment with traffic in the afternoon. We used a Bluetooth analyzer application for collecting BLE RSSI data on the smartphone. RSSI data was measured after practicing a walking speed that would allow BVI people to move 15 m in about 16 s. One set of data was collected per day. All three sets of data were collected over three days. During data collection, the intersection was a real environment in which vehicles were moving randomly. Data was stored at two samples per second for ten minutes in each zone. Therefore, 1200 samples of data of 8 RSSI values were stored, and a total of 10,800 samples of data were collected from nine zones. We had three sets of data. Thus, the total data set collected was 32,400 samples. To create a model that localized a person within each region, 80% of the data, 25,920 samples, from the first and second datasets were used for training, the remaining 20%, 6480 samples, for validation, and the remaining data from the third dataset was used for testing. This ensured fairer evaluation. Training data and validation data were selected randomly. We set the number k of kNN as 131 because k was calculated as the square root of (10,800 samples × 2 data sets × 80% training).

### 6.2. Results

**Figure 8:**
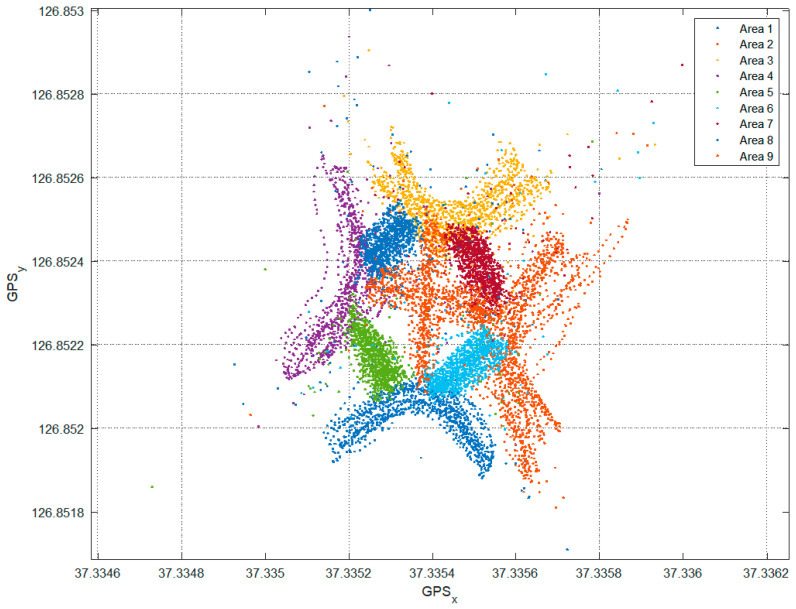
Data sampling areas as illustrated by GPS.

The RSSI values were measured in each of the nine zones determined in Figure 7, and the GPS values of the locations where the data were measured were stored. The points moved by region for data acquisition are shown in Figure 8. As with the feasibility study, outliers that deviate from the actual location due to noise mixed in the GPS data can be identified visually.

In Table 2, when the kNN classifier is used, the highest validation accuracy is the model using the average RSSI value and standard deviation as features and a moving average size of 5 s, with an accuracy of 96.63%.

In Table 3, when the SVM classifier is used, the highest validation accuracy is the model using the average RSSI value, standard deviation, median value, and IQR value and the moving average size of 10 points, with an accuracy of 98.21%.

**Figure 9:**
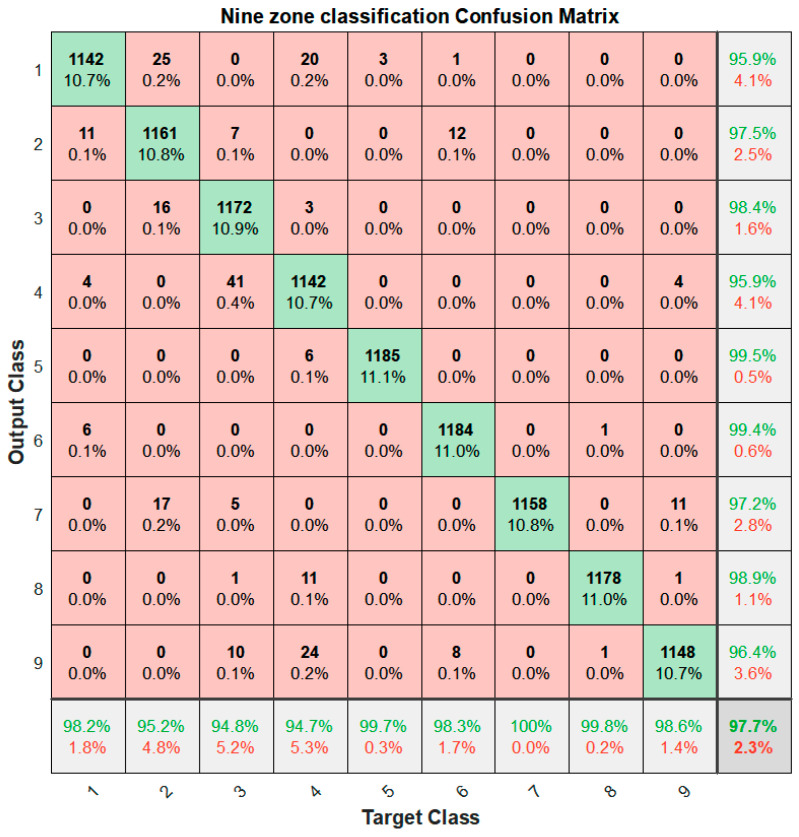
Confusion matrix for the nine-zone classification task.

When Table 2 and Table 3 were compared, the classifier had the highest accuracy with the SVM method, the number of moving averages was 10 points, and with all four features, achieved an accuracy of 98.21% on the validation set. Figure 9 shows the confusion matrix on the withheld testing set used with the trained model on the average RSSIs, standard deviation, median, and IQR of the 10-point moving average with the SVM model having the highest accuracy. The accuracy achieved was 97.7%.

The confusion matrix of Figure 9 is presented in Table 4, where five different performance estimators are reported: accuracy, specificity, sensitivity, precision, and F1 score. These parameters were evaluated for each class separately and in overall terms as follows: Accuracy = (TP + TN)/(TP + TN + FP + FN), Sensitivity = TP/(TP + FN), Specificity = TN/(TN + FP), Precision = TP/(TP + FP), F1 = TP/(TP + 0.5 × (FP + FN)) where TP stands for true positives (correct classification of data window as part of the selected class), TN are true negatives (correct classification of data window as not being part of the selected class), FP are false positives (wrong classification of data window as part of the selected class), and FN are false negatives in the classification (wrong classification of data window as not being part of the selected class). Overall accuracy was evaluated as the trace of the confusion matrix divided by the total number of classified windows [38]. Overall specificity, sensitivity, precision, and F1 score were obtained by summing TP, TN, FP, and FN values obtained for each class. The overall accuracy was 99.48% and specificity, sensitivity, precision, and F1 score were above 90% for all zones.

## 7. Discussion

### 7.1. Summary of Findings

In this work, we propose a location detection algorithm and an APS system with a built-in Bluetooth module to detect the location of BVI people when crossing outdoor intersections. Crossing a crosswalk at an intersection is one of the problems that BVI people must overcome. The intersection was divided into nine zones, allowing us to find out which zone a BVI pedestrian could potentially be in with high accuracy.

Two different machine learning classifiers, kNN and SVM, were compared to evaluate the location detection accuracy in a realistic outdoor setting. Further, the size of moving average windows was varied from three to ten samples, and six feature sets were considered. Via this experimental process, the classification model achieving the highest accuracy, along with the best set of features and window size were found. Our results showed that the system performed with the highest accuracy when using SVM, when the size of the moving average window was ten points, and when the mean, standard deviation, median, and IQR values were all used as features.

### 7.2. Integration with Useful User Interfaces

**Figure 10:**
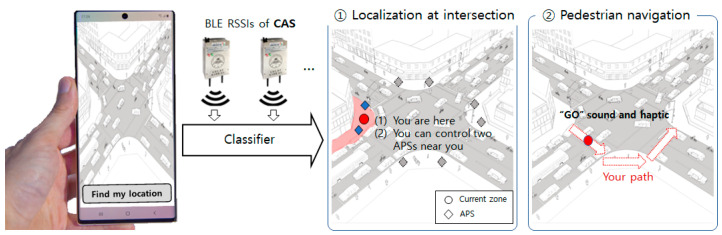
Example of CAS for pedestrian navigation after localization at the intersection.

Our method can be implemented in real-time on a smartphone and can be used in a walking path guidance system. The results enable the operation of the nearest APS based on the user to obtain information about traffic lights. Furthermore, using the proposed method, pedestrian navigation can determine whether a BVI person is waiting for a crosswalk, crossing, and when the crossing is completed. This information can help determine when walking navigation guides BVI people to the next route as shown in Figure 10.

In addition, this work complements existing research on wearable systems and on supporting BVI people [39,40,41]. The CAS system can be applied to various wearable systems that have a communication module that can receive RSSI values through Bluetooth communication instead of a smartphone or can be added. This makes it possible to identify the location of BVI pedestrians at the intersection and guide the walking route.

### 7.3. Limitations and Future Work

WiFi Fine Time Measurement (FTM) [42,43] and UWB [44] techniques have been used in localization recently. Currently, however, APS uses only BLE rather than more recent techniques. Thus, this paper focused on BLE RSSI.

In our study, the machine learning classifiers considered were limited to kNN and SVM, with a moving average method for noise filtering. Features considered included the mean RSSI value, standard deviation, median value, and IQR. Different classification models, different noise filtering methods, and other features may produce different results. In the case of surveys, we let participants choose the amount of time they can wait based on their experience, rather than how they respond after experiencing the system. Therefore, our survey results may vary if one experiences an actual system.

We measured the data in the presence of traffic. However, we did not quantify accurate information about traffic volume. Depending on the traffic conditions on the road, the results of our method may differ.

This study was to classify the zones where BVI pedestrians would be located at an intersection. It is important to classify the zone where such a person is located and operate only the acoustic signal in that zone, but it is also important to know their real location in real-time, not the zone where they are located. As this study uses a moving average, the response may be delayed or give inaccurate results while the visually impaired are moving rather than when they are stationary. To provide more information with pedestrian navigation for BVI people, in future research, we will develop an application that can measure BLE RSSI data and classify the user’s position in the smartphone. We intend to calculate actual locations at the intersection in real-time by using the BLE RSSI signal. In addition, since the moving average filter requires a waiting time to obtain enough data for averaging in the beginning, it may be possible to apply a real-time filter such as the Kalman filter to solve this problem.

Since UWB functions are increasingly being added to smartphones [45,46], if UWB is applied to APS for localization in the future, it is expected that not only will the accuracy be further improved, but also it will be more robust against noise.

Finally, as suggested in the previous subsection, further work needs to be done to better understand how to integrate such navigation data into a usable user interface, whether that is using a smartphone or additional devices such as wearable, haptic or auditory interfaces.

## 8. Conclusions

In this paper, we presented the CAS system that can detect the location of BVI pedestrians at an intersection and a localization method with a high location classification rate. RSSI signals required noise filters such as a moving average filter. However, to use the moving average filter, it is necessary to determine how many points of data should be averaged.

In the feasibility study, the intersection was divided into four sections to determine whether the developed system could recognize the pedestrian position. In addition, the location of pedestrians could be determined using RSSI signals from each APS with 99.8% accuracy with six points (three seconds’ window).

Experimentally, increasing the number of points eventually means increasing the initial waiting time. Thus, we conducted a survey to find out how long BVI people are likely to wait for results. From this survey, we found that many respondents could wait for 5 s.

To extend the feasibility study based on a five-second window maximum, we divided the intersection into nine zones, introduced another machine learning classifier to compare (SVM), and combined the moving average RSSI value, standard deviation, median, and IQR as features to the model. In addition, the window size for moving average was varied from three to ten, and we discovered which combination could produce the highest classification performance. The best performance was achieved, on a withheld test set collected on a different day, with an SVM, using a ten-points window size and the moving average of RSSIs, standard deviation, median, and IQR, as features. This CAS system could help BVI people to identify information about the nearest APS based on their location. Further, when applied to pedestrian navigation, they will be able to determine whether they are waiting, crossing, or have completed the crossing, and whether or not they go off the path that reaches their destination.

## Figures and Tables

**Table 1:** Maximum allowable waiting time according to location classification accuracy.

Waiting Time(s)	70–80%	80–90%	90–100%
Can’t use	22	11	0
1	8	10	24
2	15	23	17
3	33	30	25
4	4	3	6
5	36	40	39
6	1	1	1
7	0	0	0
8	13	14	6
9	0	0	0
10	0	0	14
Total	132	132	132

**Table 2:** The validation results of the kNN classifier of 6 inputs and several moving average points.

Number of Points Inputs	3	4	5	6	7	8	9	10
RSSI_avg_	75.62	80.86	84.99	88.24	89.77	93.61	94.05	96.35
RSSI_avg_ and SD	76.53	83.41	87.80	90.23	92.07	94.89	95.21	96.63
Median	66.57	75.99	75.46	81.12	79.92	86.75	86.31	88.80
Median and IQR	69.31	78.15	76.87	81.47	84.33	87.54	87.89	89.80
RSSI_avg_, SD, Median and IQR	74.02	81.21	82.81	87.50	88.70	92.07	92.17	94.33
RSSI_avg_ and Median	72.23	78.80	82.05	86.99	88.08	90.89	91.89	94.14

**Table 3:** The validation results of the SVM classifier of 6 inputs and several moving average points.

Number of Points Inputs	3	4	5	6	7	8	9	10
RSSI_avg_	74.44	79.01	85.76	88.66	90.54	93.03	93.84	96.09
RSSI_avg_ and SD	78.66	83.58	88.29	90.91	93.96	94.82	96.21	97.79
Median	64.07	71.68	74.11	78.59	80.78	87.33	84.66	88.75
Median and IQR	67.08	76.64	75.81	82.49	84.22	88.45	89.07	91.63
RSSI_avg_, SD, Median and IQR	77.66	84.23	87.75	91.14	93.93	95.30	97.30	98.21
RSSI_avg_ and Median	73.49	80.58	84.34	89.05	91.91	93.24	94.26	96.56

**Table 4:** Zone classification performances.

	Zone1	Zone2	Zone3	Zone4	Zone5	Zone6	Zone7	Zone8	Zone9	Overall
Accuracy (%)	99.35	99.18	99.23	98.95	99.92	99.74	99.69	99.86	99.45	99.48
Specificity (%)	99.78	99.39	99.33	99.33	99.97	99.78	100.00	99.98	99.83	99.71
Sensitivity (%)	95.89	97.48	98.40	95.89	99.50	99.41	97.23	98.91	96.39	97.68
Precision (%)	98.19	95.24	94.82	94.69	99.75	98.26	100.00	99.83	98.63	97.68
F1	97.03	96.35	96.58	95.29	99.62	98.83	98.60	99.37	97.49	97.68
